# Development of coating formulation with silica–titania core–shell nanoparticles against pathogenic fungus

**DOI:** 10.1098/rsos.180633

**Published:** 2018-08-15

**Authors:** Jaya Verma, Arpita Bhattacharya

**Affiliations:** Amity Institute of Nanotechnology, Amity University, Noida, Uttar Pradesh 201303, India

**Keywords:** silica–titania core–shell nanoparticle, peptization process, antifungal effect, pathogenic fungus, polyurethane

## Abstract

In the present study, we developed an antifungal coating formulation using silica, titania and silica–titania core–shell nanoparticles individually. The idea behind the synthesis of core–shell nanoparticles was to use the mechanical strength of silica and the antimicrobial property of TiO_2_ together. These nanoparticles were characterized by dynamic light scattering, transmission electron microscopy, scanning electron microscopy, EDX, FTIR and X-ray diffraction. Silica nanoparticles of 92 nm were prepared by the sol–gel process, while TiO_2_ nanoparticles and nano-core–shells were prepared through the peptization process with a size of 77 and 144 nm separately. The antifungal effect of the prepared nanoparticles was observed in potato dextrose agar media using the concentration of nanoparticles at 1 wt%. These nanoparticles were incorporated in two types of binder, polyurethane and polyacrylic, with the same concentration of nanoparticles. Coatings were applied on tiles, dried and tested against pathogenic fungus, and fungus growth reduction was observed up to 7–10 days. Coatings developed with TiO_2_ nanoparticles have shown good growth reduction of pathogenic fungus, but coatings formulated with silica–titania core–shell nanoparticles killed the fungus fusarium completely and have shown around 90% growth reduction for acremonium species also.

## 1. Introduction

Indoor air pollution is a serious public health concern and a major cause of morbidity and mortality worldwide. The presence of microbial populations in damp indoor environments is one of the main causes of the degradation of indoor air quality and contributes to sick building syndrome [1–5]. Microorganisms may produce contaminants, i.e. aerial particles, such as spores, allergens, toxins and other metabolites, that can be serious health hazards to occupants. A number of researchers have already pointed out that indoor building materials can become major sites of microbial growth when promoting conditions, such as high humidity and nutrient content, are present [6–8]. These conditions are easily satisfied in water-damaged buildings, damp buildings and badly insulated buildings. Results from earlier studies have revealed that various microorganisms, including potentially pathogenic species like *Acremonium kiliense*, *Acremonium strictum* and *Fusarium solani* etc., are detected on building materials [9–11].

*Acremonium* species are pathogenic fungus that cause disease in humans. Mold & Bacteria Consulting Laboratories (MBL) have proved this. Infection with *Acremonium* has been described in immunocompromised patients. It can cause fungal maxillary sinusitis. In the medical literature, it has been reported as the cause of pulmonary infections and infections of the cornea and nails in individuals with weak immune systems. The most common species in indoor environments are *A. strictum*. In indoor environments, *Acremonium* species are primarily isolated from acoustic and thermal fibreglass insulation used in heating ventilation and air-conditioning systems, cooling coils, drain pans, windowsills and water from humidifiers. Also found on carpet and mattress dust, damp or wet walls (especially in basements), gypsum board and wallpaper. *Fusarium* species also cause a broad spectrum of infections in humans, including superficial infections such as keratitis and onychomycosis, as well as locally invasive and disseminated infections. Invasive and disseminated infections occur almost exclusively in severely immunocompromised patients, particularly among those with prolonged and profound neutropenia and severe T-cell immunodeficiency. Among patients with haematologic malignancy, the infection predominates during periods of neutropenia, typically among patients with leukaemia receiving induction chemotherapy*. Fusarium* also causes various diseases on cereal grains and occasionally causes infection in animals [12–14].

A substantial amount of literature has been published on the antimicrobial effect of TiO_2_ nanoparticles [15,16]. Its photocatalytic process in water is effective against a wide range of organisms, such as algae, viruses, fungi and bacteria. It should be noted that the different tests were carried out in aqueous slurry or with aqueous inoculum (sprayed or dropped), emphasizing the major role of water in the microorganism photo-killing process. In addition, TiO_2_ nanoparticles can be used as (i) a powder, usually dispersed in aqueous slurry or (ii) a film/coating applied to various substrates. Several works have highlighted very high bactericidal efficiency on different microorganisms, but there is no detailed explanation for antifungal testing with core–shell nanoparticles [17].

In this paper, we formulated silica, titania and silica–titania core–shell nanoparticles individually. Silica is a very hard natural material having high scratch resistance. Nano-silica has been proved to be a very promising material due to its low density, good thermal and mechanical stability, and chemical inertia in many fields of applications like catalysis, drug delivery, chemical sensors, chromatography, micro-reactor and biological images [18]. Coating of titania onto silica can enhance its stability and UV absorption property because individual TiO_2_ nanoparticles with high surface area are thermally unstable and lose their surface area readily [19]. This core–shell formation improves the antimicrobial property of the coatings as well as strength of the coated surface. Coating formulations were developed in polyurethane (provides strong bonding/improves the scratch-resistant property) and polyacrylic (high durability implies resistance to the effects of UV degradation from the sun) binder that also supports in reducing fungus growth [20–22]. In this study, the anti-scratch property of these coatings developed with silica and silica–titania core–shell nanoparticles in polyurethane was tested at different loads from 3 to 9 N using the Universal Material Tester (CETR UMT-3). This formulation is also cost-effective. In this paper, the peptization process was used for preparation of core–shell nanoparticles because this gives better particle size at low processing temperature.

## 2. Experimental

### 2.1. Materials

Tetraethyl orthosilicate (TEOS) and titanium tetra-isopropoxide (TTIP) were purchased from Sigma-Aldrich Chemicals Pvt Ltd (India), ethanol from Merck (India) and ammonium hydroxide from Qualikems Fine Chemical Pvt Ltd (India). Nitric acid was purchased from Fisher Chemicals (India). Potato dextrose agar (PDA) was purchased from HiMedia Laboratories Pvt Ltd (India), and polyurethane and polyacrylic were purchased from Dalton Chemicals Pvt Ltd (India).

### 2.2. Methods

#### 2.2.1. Preparation of silica nanoparticles through the sol–gel method

TEOS was used as a precursor for synthesis of SiO_2_ nanoparticles. Nano-silica was prepared from 8 ml of TEOS in 100 ml of ethanol and 35 ml of DI water mixture. This solution was stirred for 40 min and after this ammonium hydroxide was added drop-wise to maintain pH at 10. This solution was kept for 24 h and then centrifuged at 8000 r.p.m. for 5–10 min. After centrifugation, the material was dried at 100°C overnight and calcinated at 650°C for 2 h.

#### 2.2.2. Preparation of titania nanoparticles through the peptization method

In a typical peptization process, a specific amount of titanium isopropoxide (2 ml) was added to 50 ml of distilled water under stirring. Within a few minutes, a white suspension was formed and then coagulated titanium oxide was precipitated. To this titania gel, 1 ml of 70% nitric acid was added when the acid concentration maintained in the reaction media became about 0.20 M. The resulting suspension was stirred for 4 h at a temperature of 70°C. After this, the whole material was dried overnight at 70°C.

#### 2.2.3. Preparation of silica–titania core–shell nanoparticles using the peptization process

**Figure 1.**
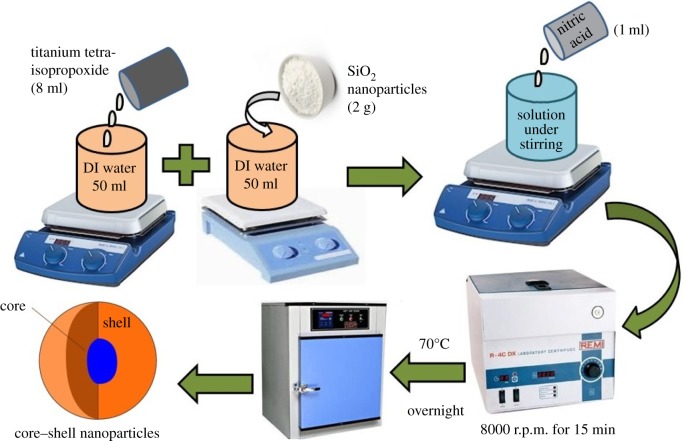
Preparation of SiO_2_–TiO_2_ core–shell nanoparticles.

For preparation of silica–titania core–shell nanoparticles through the peptization process, TTIP (2 ml) was added to 50 ml of distilled water under stirring in the presence of 2 g of silica nanoparticles prepared through the sol–gel process as mentioned in §2.2.1. A 1 ml aliquot of 70% nitric acid was added into this (when the acid concentration maintained in the reaction media became about 0.20 M) and stirred for 4 h at 70°C. After this, whole material was centrifuged at 8000 r.p.m. for 10–15 min and dried at 70°C for overnight (figure 1).

#### 2.2.4. Coating formulation

Coating formulations of silica, titania and silica–titania core–shell nanoparticles were developed with polyurethane and polyacrylic binders individually at 1 wt% concentration of materials on tiles for antifungal testing. The same coatings were developed on a glass substrate (2 × 2 cm) for scratch testing analysis, with a coating thickness of 100 µm. These coatings were developed using a paint brush and dried in an oven at 100°C for 1 h.

#### 2.2.5. Antifungal testing procedure

Antifungal testing was performed by dissolving 39 g of PDA in 1000 ml of distilled water under stirring. This solution was sterilized by autoclaving at 15 psi at 121°C for 15 min. Nanoparticle concentration was maintained in the media at 1 wt%. This was cooled down to room temperature prior to dispensing. The fungi were inoculated on separate Petri plates containing sterilized media. These Petri plates placed in a biochemical oxygen demand (BOD) chamber. The same procedures were followed for antifungal testing of coated tiles developed with polyacrylic and polyurethane with the same nanoparticle concentration. Growth of fungus was observed up to 7–10 days.

#### 2.2.6. Anti-scratch testing procedure

Anti-scratch testing of a coated glass substrate was performed using UMT CETR Unit-3 from load 3 to 9 N. It has a high-density cast iron vibration-dampened frame. Position resolution was maintained at 1 µm for the lateral positioning system and 2 µm for the vertical positioning system. Encoder resolution was maintained at 0.25 µm for the lateral positioning system and 0.5 µm for the vertical positioning system. Scratch was applied by different loads (3–9 N) of 9 mm scratch length on the coated glass substrate. After that, analysis of the scratched samples was performed by scanning electron microscopy (SEM) at a magnification of 150×.

#### 2.2.7. Toxicity study of coated samples

A toxicity test was simply performed in DI water of polyurethane (PU)-coated substrate/tiles containing silica–titania core–shell nanoparticles of 1 wt% concentration. In two separate test tubes, 10 ml of water was taken. Polyurethane-coated sample was added in one test tube, which was kept for 15 days. Afterwards, UV visible analysis of these samples was carried out.

## 3. Characterization

The particle size of the prepared nanoparticles was measured in double distilled water by sonication through dynamic light scattering (DLS) using Malvern instruments (Zetasizer Nano S-90). Fourier transform infrared (FTIR) spectroscopy was performed with a Shimadzu 8400 spectrophotometer. X-ray diffraction was carried out using a Bruker D8 Focus. FEI Tecnai G2 F20S was used for transmission electron microscopic (TEM) analysis of nanoparticles in the dry state and SEM was performed using a Zeiss scanning electron microscope. Element identification was carried out by a Bruker EDX analyser. UV–Vis analysis of samples was performed with a UV 1800 Shimadzu UV spectrophotometer. The antifungal effect was observed in PDA media. A Universal Materials Tester (CETR-UMT 3) was used for scratch testing analysis.

## 4. Results and discussion

**Figure 2.**
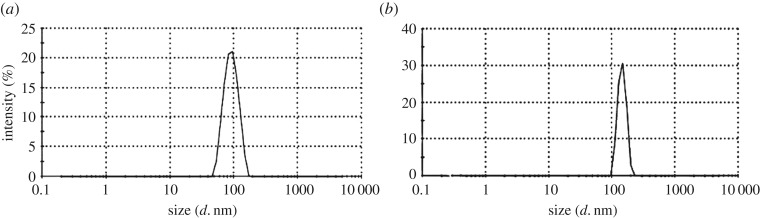
DLS of (*a*) silica nanoparticles and (*b*) silica–titania core–shell nanoparticles.

Particle size of the prepared core–shell nanoparticles was observed to be 144 nm with a silica particle size of 92 nm that shows a thin coating of the TiO_2_ layer on the silica core (26 nm) (figure 2). Even the titania nanoparticle prepared individually had a particle size of 77 nm.

**Figure 3.**
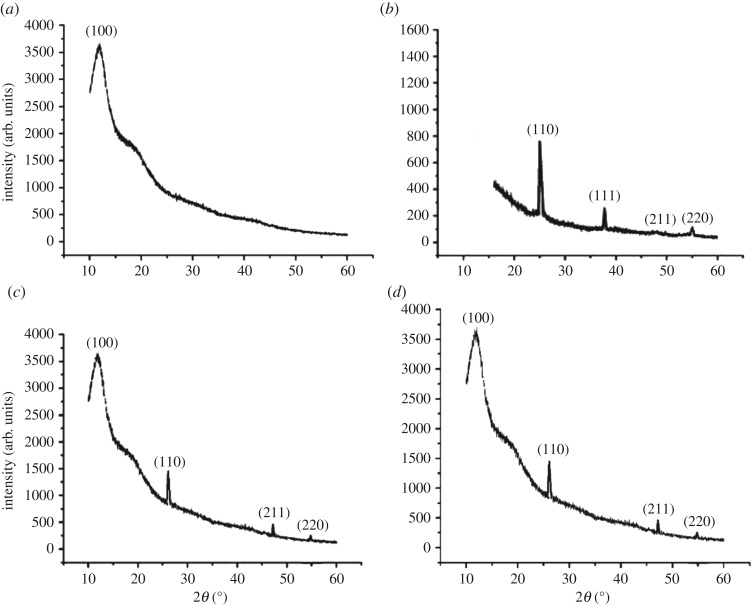
XRD analysis of (*a*) nano silica, (*b*) nano titania, (*c*) polyacrylic-coated substrate containing silica–titania core–shell nanoparticles and (*d*) polyurethane-coated substrate containing silica–titania core–shell nanoparticles.

X-ray diffraction in figure 3 also confirms the formation of core–shell nanoparticles. The silica nanoparticle shows a broad peak (amorphous nature) at 2*θ* = 15*°* (*hkl* = 100) and the titania nanoparticle shows sharp peaks (crystalline nature) at 2*θ* = 25*°*, 37*°*, 46*°*, 55*°* (*hkl* = 110, 111, 211, 220). In polyurethane and polyacrylic-based nano-core–shell XRD, one broad peak was obtained at 2*θ* = 15*°* (*hkl* = 100), which shows the presence of the core material silica and other sharp peaks at 2*θ* = 25*°*, 46*°*, 55*°* (*hkl* = 110, 211, 220), which indicates the presence of TiO_2_ nanoparticles.

**Figure 4.**
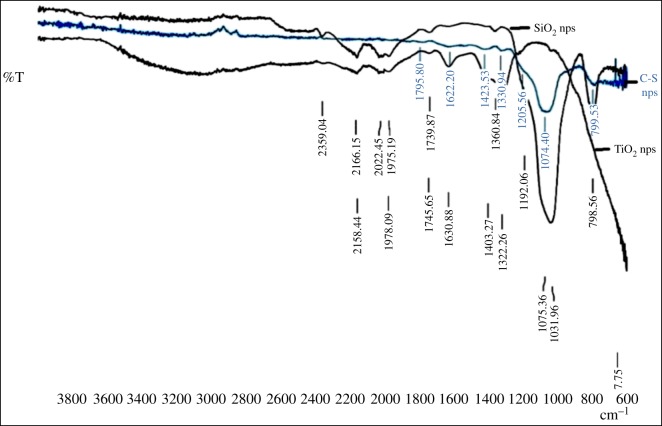
FTIR analysis of core–shell nanoparticles with silica and titania nanoparticles.

In FTIR analysis, figure 4 shows the FTIR spectra of silica, titania and silica–titania core–shell nanoparticles. In the spectra of silica, the band around 1070 cm^−1^ corresponds to assymetric stretching vibration of the Si–O–Si bond, whereas the 3300 and 1640 cm^−1^ bands have appeared for H–O–H stretching and bending of absorbed water. Another peak at around 910 cm^−1^ corresponds to the Si–OH bond. In the case of SiO_2_@TiO_2_ spectra, along with the peaks of SiO_2_ spectra, a band at around 950 cm^−1^ appeared for the Si–O–Ti bond, which supports formation of a layer of titania on a silica core.

**Figure 5.**
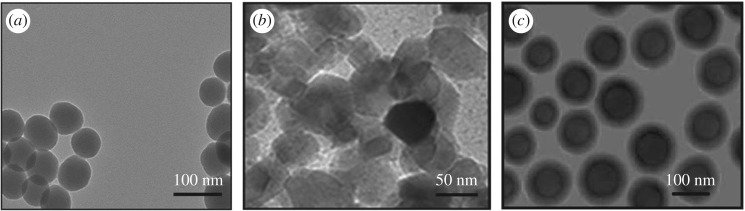
TEM images of (*a*) silica nanoparticles, (*b*) titania nanoparticles and (*c*) silica–titania core–shell nanoparticles.

TEM analysis was carried out of prepared silica, titania and silica–titania core–shell nanoparticles to confirm the formation of a core–shell structure with a particle size of around 100 nm in spherical shape as shown in figure 5. This analysis was performed at 20 kV and 100× magnification, with a point resolution of 0.27 nm. In this analysis, TEM revealed the primary particle size of the core–shell nanoparticle. Previous measurement of particle size through DLS was carried out to determine the true state of particles in media, i.e. around 144 nm. The difference in particle size of core–shell measurement through DLS was because the measurement was carried out in the liquid state and due to the solvent molecules associated with particles, showing a larger size of particles when compared with TEM analysis.

**Figure 6.**
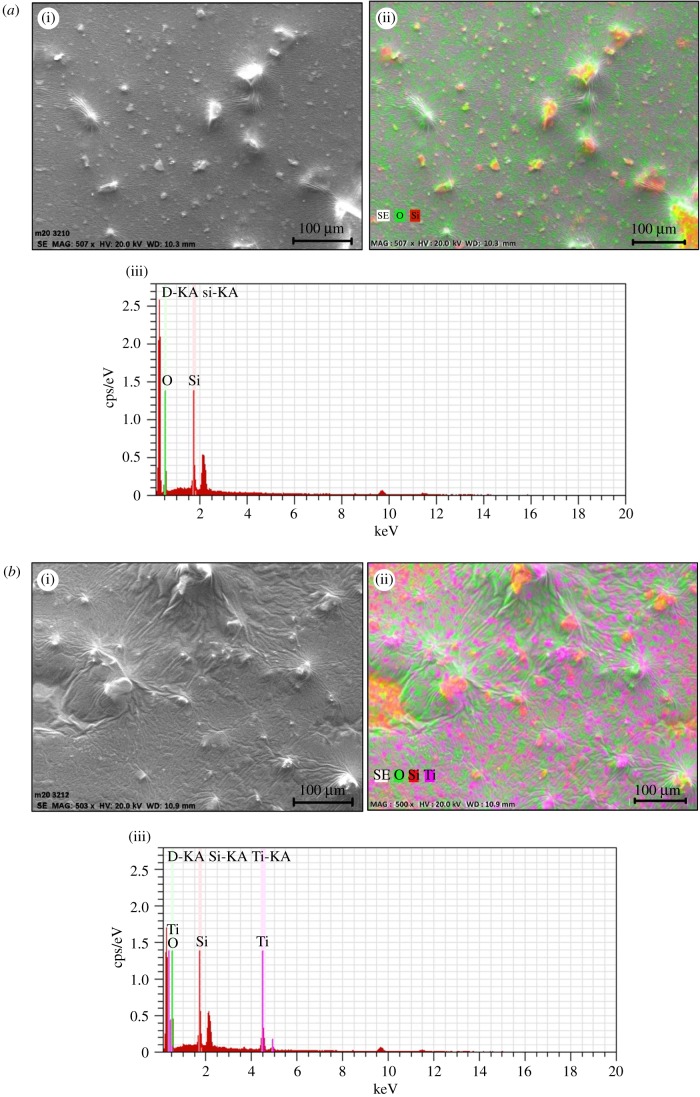
(*a*) (i,ii) SEM image of SiO_2_-PU coating and (iii) EDX analysis of SiO_2_-PU nanocoating. (*b*) (i,ii) SEM image of core@shell-PU nanocoating and (iii) EDX analysis of core@shell-PU nanocoating.

**Figure 7.**
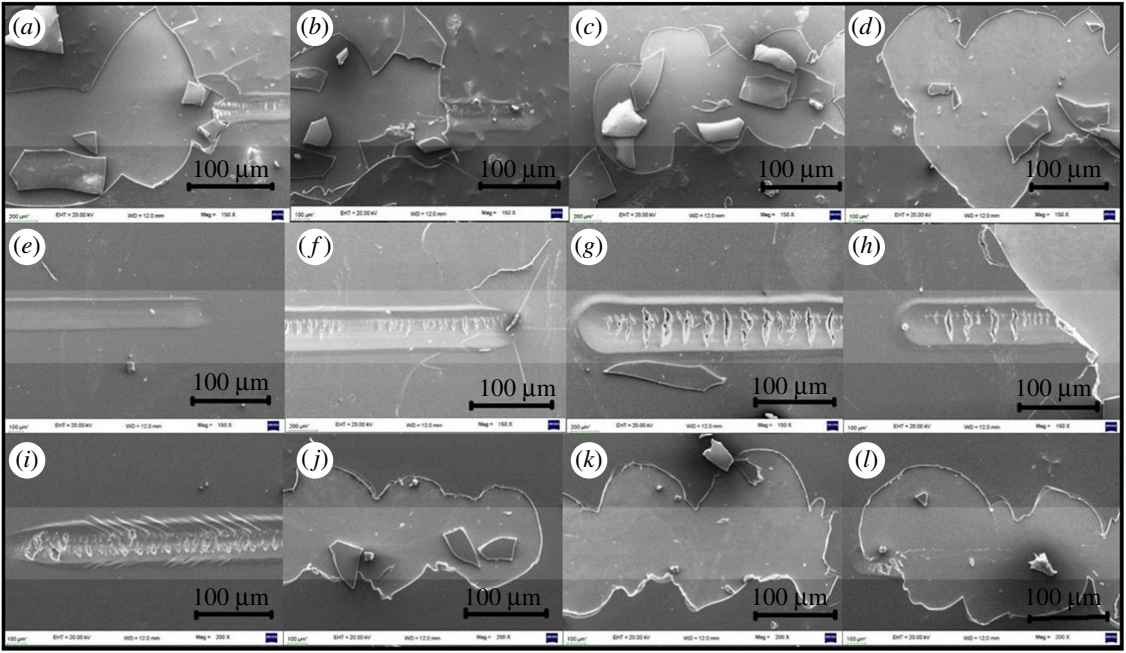
SEM images at different loads: (i) scratched polyurethane coating (*a*) 3 N, (*b*) 5 N, (*c*) 7 N and (*d*) 9 N; (ii) scratched polyurethane-coating containing silica nanoparticles (*e*) 3 N, (*f*) 5 N, (*g*) 7 N and (*h*) 9 N and (iii) scratched polyurethane coating with silica–titania core–shell nanoparticles (*i*) 3 N, (*j*) 5 N, (*k*) 7 N and (*l*) 9 N.

SEM analysis of all prepared coatings was carried out for surface topography of this core–shell nano-coating formulation and confirmation of elements present in this core–shell was done by EDX analysis (figure 6). Anti-scratch property was also analysed from SEM for applied loads from 3 to 9 N on a glass-coated substrate containing silica and silica–titania core–shell nanoparticles in polyurethane (figure 7).

**Figure 8.**
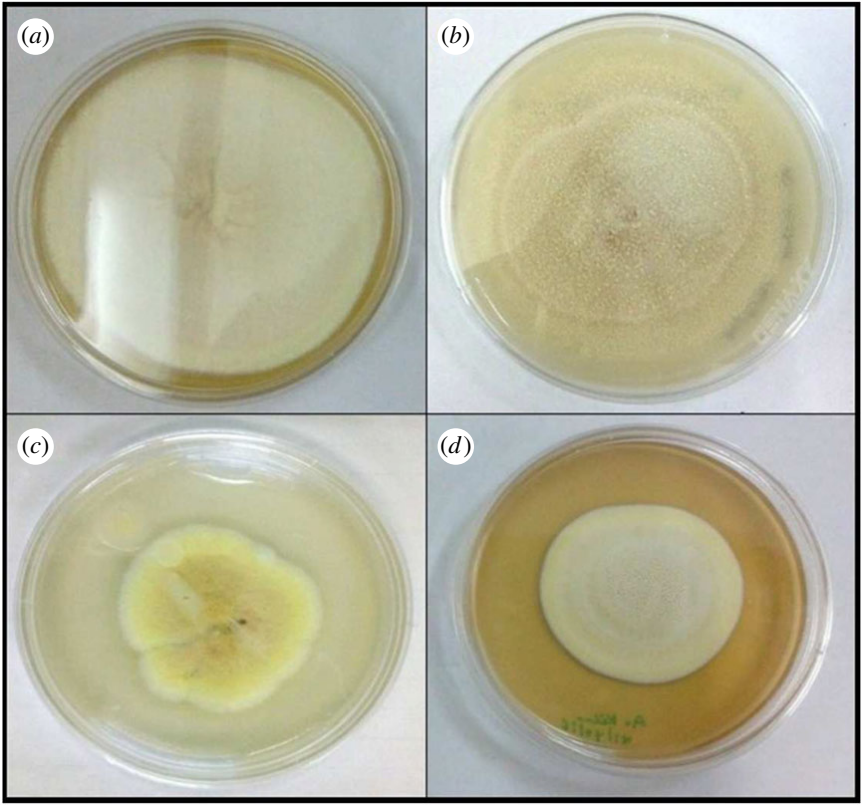
Growth of *Acremonium kiliense* in (*a*) control, (*b*) media containing silica nanoparticles, (*c*) media containing titania nanoparticles and (*d*) media containing silica–titania core–shell nanoparticles.

**Figure 9.**
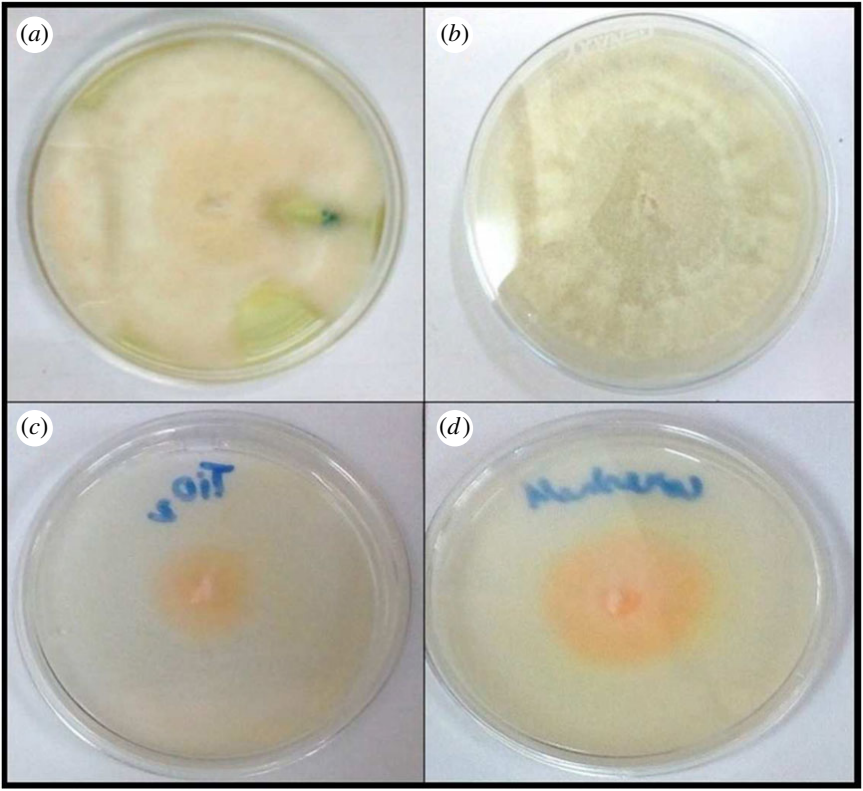
Growth of *Acremonium strictum* in (*a*) control, (*b*) media containing silica nanoparticles, (*c*) media containing titania nanoparticles and (*d*) media containing silica–titania core–shell nanoparticles.

**Figure 10.**
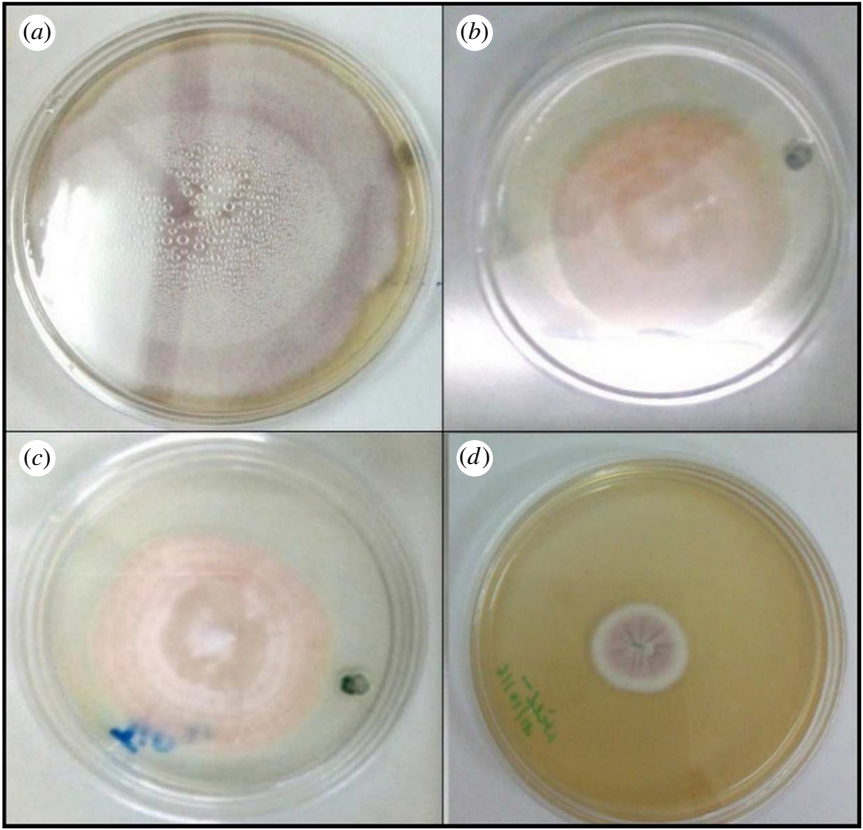
Growth of *Fusarium solani* in (*a*) control, (*b*) media containing silica nanoparticles, (*c*) media containing titania nanoparticles and (*d*) media containing silica–titania core–shell nanoparticles.

Antifungal testing of prepared nanoparticles was carried out against *A. kiliense*, *A. strictum* and *Fusarium solani*; as we can see from the results in figures 8–10, the presence of nanoparticles reduces the growth of fungus. Growth reduction was observed up to 70% in the presence of TiO_2_ nanoparticles, but silica–titania core–shell nanoparticles have shown the best result against *F. solani*. The growth reduction percentages of fungus in the presence of all prepared nanoparticles are presented in table 1.

**Table 1.** Fungus growth analysis of all prepared nanoparticles.

fungus	material (nanoparticles 1 wt%) in media	fungal growth reduction due to the presence of nanoparticles (%)
*Acremonium kiliense*	silica nanoparticle	27.5
	titania nanoparticle	42.5
	core–shell nanoparticle	43
*Acremonium strictum*	silica nanoparticle	21.48
	titania nanoparticle	70
	core–shell nanoparticle	50.1
*Fusarium solani*	silica nanoparticle	37.5
	titania nanoparticle	44
	core–shell nanoparticle	80

**Figure 11.**
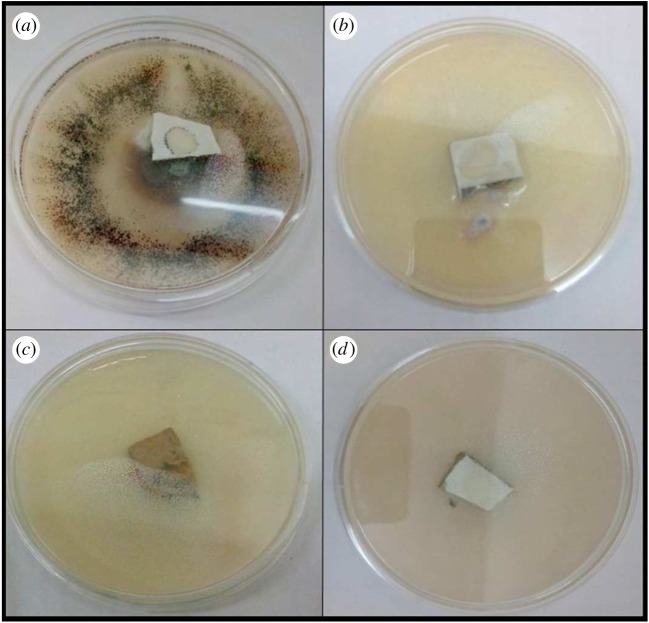
Growth of *Fusarium solani* in (*a*) tiles coated with pure PU, (*b*) tiles coated with PU containing silica nanoparticles, (*c*) tiles coated with PU containing titania nanoparticles and (*d*) tiles coated with PU containing silica–titania core–shell nanoparticles.

**Figure 12.**
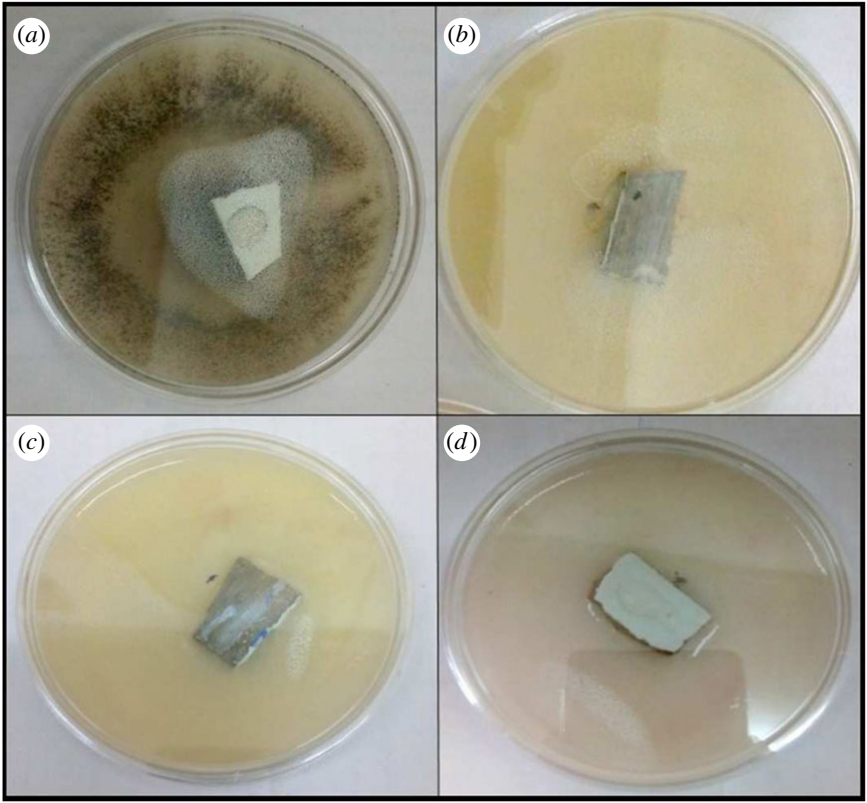
Growth of *Fusarium solani* in (*a*) tiles coated with pure polyacrylic (PA), (*b*) tiles coated with PA containing silica nanoparticles, (*c*) tiles coated with PA containing titania nanoparticles and (*d*) tiles coated with PA containing silica–titania core–shell nanoparticles.

**Graph 1.**
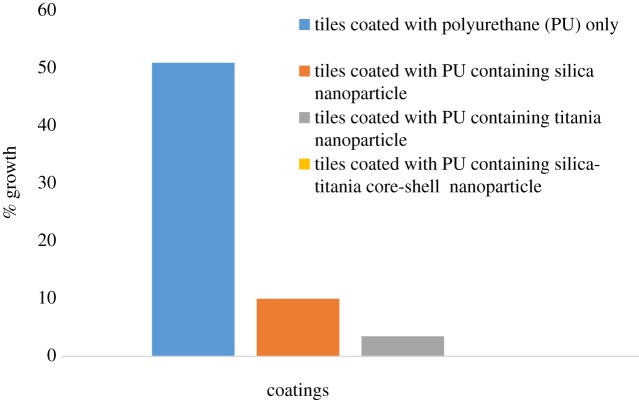
Graphical analysis of polyurethane-based anti-fungal coatings.

**Graph 2.**
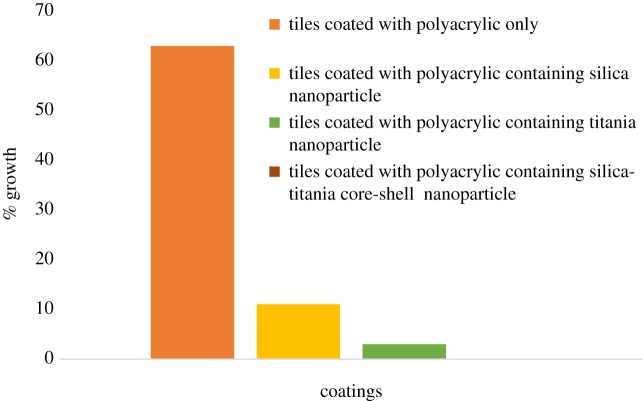
Graphical analysis of polyacrylic-based anti-fungal coatings.

Coatings developed with polyurethane and polyacrylic were tested against pathogenic fungus and it was found that the presence of silica–titania core–shell nanoparticles was the most effective for antifungal testing, as illustrated in table 2; moreover, these coating formulations have shown the best antifungal result against *F. solani.* As shown in figures 11 and 12, the coating formulation with core–shell nanoparticles killed the fungus completely, and the observed growth analysis revealed a rate of up to 0% for the polyurethane-based coating and 0.01% for the polyacrylic-based coating containing silica–titania core–shell nanoparticles (graphs 1 and 2).

**Table 2.** Fungus growth analysis of developed coating formulations.

fungus	coatings (nanoparticles 1 wt%)	fungal growth analysis of polyurethane-based coatings (%)	fungal growth analysis of polyacrylic-based coatings (%)
*Acremonium kiliense*	pure polymer	60	65.2
	silica nano-coating	25.5	27
	titania nano-coating	15.3	16.5
	core–shell nano-coating	12.5	13.1
*Acremonium strictum*	pure polymer	57.7	63.9
	silica nano-coating	24.98	25.5
	titania nano-coating	14.9	15.2
	core–shell nano-coating	8.5	9.1
*Fusarium solani*	pure polymer	51	63
	silica nano-coating	10	11
	titania nano-coating	3.5	3.4
	core–shell nano-coating	0	0.01

This indicates a more favourable result with use of these nanoparticles in nano-coating application with different types of binder to reduce fungus growth as well as to improve the anti-scratch property. This core–shell formation provides more surface area to kill fungus and improved overall particle stability and dispersibility of the coating system. One more advantage of this formulation is that it is cost-effective when compared with a pure TiO_2_ nano-coating formulation.

**Figure 13.**
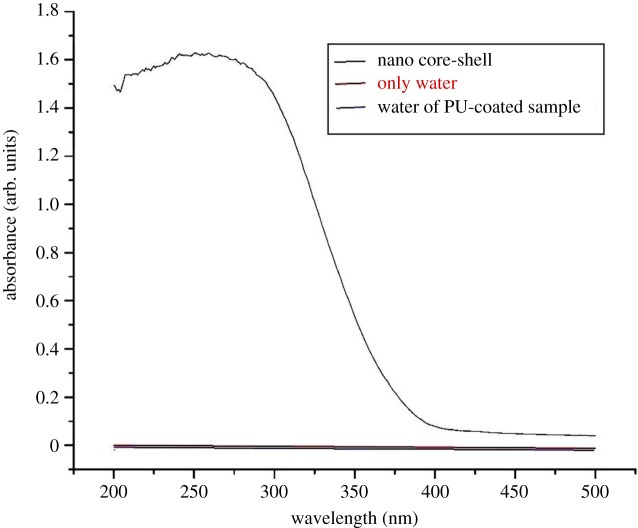
Toxicity study of polyurethane-coated substrate containing silica–titania core–shell nanoparticles.

In the toxicity study of the coatings formulated, as shown in figure 13, the UV–Vis spectrum of the nano SiO_2_@TiO_2_ core–shell has shown an absorbance peak at around 300 nm, but there was no peak for water in which the PU-coated sample was placed for 15 days, being the same as that for a pure water sample, which means that there was no release of nanoparticles from the coating in water. So, these coatings are not showing any toxic effect in water. We can use these antimicrobial coatings in swimming pools, hospitals and toilets.

## 5. Conclusion

We successfully prepared silica, titania and silica–titania core–shell nanoparticles. Characterization with the use of DLS, X-ray diffraction, FTIR, TEM, SEM, EDX and antifungal testing was successfully carried out. Coating formulations was successfully developed with polyurethane and polyacrylic binder. Antifungal testing of these coating formulations were successfully performed against pathogenic fungus. Coatings developed with silica–titania core–shell nanoparticles in polyurethane have shown the best antifungal result against *F. solani*.

## Data accessibility

This article does not contain any additional data.
